# Therapeutic Modulation of *Demodex* Density via Isotretinoin: Insights From a Prospective Dermatological Investigation

**DOI:** 10.1111/jocd.70249

**Published:** 2025-05-28

**Authors:** Ahmed Muayad Jirjees Chakmakchi, Emine Tugba Alatas, Aslan Yurekli, Ceyda Tetik Aydoğdu, Suzan Demir Pektas

**Affiliations:** ^1^ Department of Dermatology Mugla Sitki Kocman University Faculty of Medicine Mugla Turkey

**Keywords:** acne vulgaris, *demodex*, demodicosis, isotretinoin, rosace

## Abstract

**Aim:**

*Demodex* spp. are ectoparasites that reside in pilosebaceous units, particularly on the face where sebum secretion is prominent. The sebum‐reducing effects of isotretinoin play a crucial role in the management of acne vulgaris and rosacea. This study aims to assess the effect of isotretinoin on *Demodex* density in patients with acne vulgaris and rosacea accompanied by demodicosis. The evaluation includes *Demodex* mite density, before, during, and after 6 months of isotretinoin treatment.

**Materials Methods:**

The study included patients diagnosed with acne vulgaris and rosacea accompanied by demodicosis who were prescribed isotretinoin treatment. Demographic data were collected, and *Demodex* spp. mites were detected using superficial skin biopsy before treatment initiation. Follow‐up samples were obtained at 2 and 6 months. Statistical analysis was performed using SPSS version 23, with a significance level set at *p* < 0.05.

**Results:**

A total of 36 patients, 25 with acne and 11 with rosacea, were included in our study. *Demodex* density was assessed before treatment, at 2 months, and at 6 months. Our findings showed a significant decrease in *Demodex* density with isotretinoin treatment, especially in relation to the decrease in sebum secretion (*p* < 0.001).

**Conclusion:**

This study makes a notable contribution to the existing literature by being the first to assess the impact of isotretinoin treatment on *Demodex* density. Our results indicate that isotretinoin effectively reduces *Demodex* density, likely due to a reduction in sebum production. To further explore isotretinoin's effects, future research should involve larger sample sizes and extended follow‐up periods. These findings enhance our understanding of isotretinoin's role in managing *Demodex*‐related dermatological conditions.

## 1 Introduction

The presence of *Demodex* spp. mites, specifically *Demodex* folliculorum and *Demodex* brevis, in high numbers on the human skin can lead to Demodicosis, a condition often associated with various dermatological issues. Among the most common conditions linked to Demodicosis are rosacea, acne vulgaris, and seborrheic dermatitis [1]. These mites are thought to play a role in the development or exacerbation of these skin disorders. Recent studies have highlighted the potential involvement of *Demodex* mites in other skin conditions such as perioral dermatitis and granulomatous rosacea, which are less commonly seen but can still be influenced by the presence of these ectoparasites [2, 3]. Research on the subject has been growing, further emphasizing the connection between *Demodex* infestations and skin manifestations [4, 5, 6].

Isotretinoin is a well‐established treatment for both acne vulgaris and acne rosacea, with its effectiveness in these conditions stemming from multiple mechanisms. In acne vulgaris, isotretinoin works primarily by suppressing sebum production, promoting sebocyte apoptosis (cell death), and reducing the inflammatory response in the skin. In the case of acne rosacea, isotretinoin's benefits are attributed to its ability to decrease sebocyte proliferation, reduce sebum production, minimize inflammation, and improve telangiectasias (visible blood vessels) on the skin. These actions help to manage and alleviate the symptoms associated with rosacea, making isotretinoin an important therapeutic option for patients with both conditions [7].

In our study, we aimed to evaluate the effect of isotretinoin on *Demodex* parasites in patients with acne vulgaris and rosacea accompanied by demodicosis. Within the scope of the study, it is planned to evaluate the *Demodex* mite density, before starting isotretinoin treatment, during treatment, and at 6 months of treatment. This study could provide valuable insights into the effects of isotretinoin on *Demodex* populations and its potential benefits in treating both acne vulgaris and rosacea, particularly in cases complicated by demodicosis.

## 2 Materials and Methods

The study was conducted at the Dermatology Clinic of Muğla Sıtkı Koçman University Training and Research Hospital between January 1, 2024 and July 30, 2024. The study was designed as an observational prospective study. Ethical approval for the thesis was obtained from the Muğla Sıtkı Koçman University Ethics Committee, with decision number 123, dated December 21, 2023.

### 2.1 Study Design

A total of 36 patients who presented to the Dermatology Clinic of Muğla Sıtkı Koçman University Training and Research Hospital and met the necessary inclusion criteria were enrolled in the study. Written informed consent was obtained from all participants. *Demodex* measurements were performed on the study subjects prior to the initiation of isotretinoin treatment with 20 mg/kg/day dosing and at the second and sixth months of treatment. Patients did nothave any additional topical or systemic treatments targeting *Demodex* used during the study period. Demographic data (age, gender) of the patients were also recorded.

### 2.2 Inclusion Criteria

1. Participants must be over 18 years of age.
2. Participants must be under 65 years of age.
3. Participants must have a diagnosis of demodicosis.
4. Participants must have acne vulgaris and/or acne rosacea for which isotretinoin treatment is indicated.
5. Participants must provide written consent to participate in the study.

### 2.3 Exclusion Criteria

1. Participants under 18 years of age.
2. Participants over 65 years of age.
3. Participants who have not been diagnosed with demodicosis.
4. Participants who have received antiparasitic treatment and topical/systemic treatments within 4 weeks prior.
5. Participants who have not regularly attended follow‐up visits for treatment evaluation.

### 2.4 Assessment of *Demodex* Density

*Demodex* density was evaluated prior to treatment and at the second and sixth months of therapy at the Dermatology Intervention Room of Muğla Sıtkı Koçman University Training and Research Hospital using the Standardized Skin Surface Biopsy (SSSB) method. Initial examination for the presence of *Demodex* mites was conducted using a UV dermoscope to identify clinically suspicious areas for sampling. The selected area was then cleaned with alcohol to ensure it was dry and free of contaminants.

A 1 cm^2^ area was marked on one side of a microscope slide, and a few drops of cyanoacrylate adhesive were applied to the opposite side. The slide was then firmly pressed onto the target area for approximately 1 min. After removal, immersion oil was applied, and a coverslip was placed over the sample.

The UV dermoscope was used solely to guide the selection of the most representative sampling site during the initial assessment. At the second and sixth month follow‐up visits, samples were consistently collected from the same anatomical location identified at baseline—specifically, the right cheek for all participants who did not show visible signs under UV dermoscopy.

Slides were examined under a standard light microscope at ×10 and ×40 magnifications. The number of mites present within the defined 1 cm^2^ area was counted. A count of five or more mites per cm^2^ was considered indicative of demodicosis. Based on pretreatment *Demodex* spp./cm^2^ density, patients were categorized into three groups: mild (5–9 mites/cm^2^), moderate (10–20 mites/cm^2^), and severe (≥ 21 mites/cm^2^) [8, 9].

### 2.5 Statistical Analyses

All statistical analyses were performed using IBM SPSS Statistics for Windows, Version 25.0 (Released 2017; Armonk, NY: IBM Corp.). The Shapiro–Wilk test was used to assess the normality of numerical variables. As the data did not meet the assumptions of normal distribution, nonparametric statistical methods were applied.

Changes in *Demodex* density over time (baseline, second month, and sixth month) were analyzed using the Friedman test for repeated measures. When significant overall differences were detected, post hoc pairwise comparisons were conducted using the Dunn test with Bonferroni correction. Comparisons between independent groups (e.g., acne vs. rosacea) were made using the Kruskal–Wallis test and, where appropriate, the Mann–Whitney *U* test.

The relationships between categorical variables were examined using Fisher's exact test. Correlations between numerical variables were assessed using Spearman's rank correlation analysis. A *p*‐value < 0.05 was considered statistically significant for all tests.

## 3 Results

### 3.1 Demographics of Study Participants

Among the participants in the study, 61.1% were female and 38.9% were male, resulting in a female‐to‐male ratio of 1.5. The age range of the participants was between 18 and 59 years, with an average age of 25.6 years.

#### 3.1.1 Acne Vulgaris Group

The acne vulgaris group consisted of 14 females and 11 males, with an average age of 21.7 years.

#### 3.1.2 Rosacea Group

The rosacea group included eight females and three males, with an average age of 34.6 years.

### 3.2 Classification of *Demodex* Severity

The patients in our study were classified into three groups based on *Demodex* density: mild (5–9), moderate (10–20), and severe (≥ 21). Additionally, each patient was categorized according to their clinical condition (acne or rosacea). Changes in *Demodex* density were assessed at three time points: before treatment, at 2 months, and at 6 months. Mild, expected side effects (e.g., cheilitis, dry skin) were observed, but no participants discontinued treatment due to adverse effects.

Before the initiation of isotretinoin treatment, 58.3% of patients had mild *Demodex* density (5–9), 16.7% had moderate *Demodex* density (10–20), and 25% had severe *Demodex* density (≥ 21). At the second month of treatment, 63.9% of patients exhibited a *Demodex* density of 0–4, 5.6% had mild *Demodex* density (5–9), 16.7% had moderate *Demodex* density (10–20), and 13.9% had severe *Demodex* density (≥ 21). By the sixth month of treatment, 69.4% of patients had a *Demodex* density of 0–4, 13.9% had mild *Demodex* density (5–9), 8.3% had moderate *Demodex* density (10–20), and 8.3% had severe *Demodex* density (≥ 21).

### 3.3 Evaluation of Clinical Status Based on *Demodex* Density

*Demodex* density during the pre‐treatment period exhibited a significant difference between acne and rosacea patients (Pearson Chi‐Square test, *p* = 0.017; Fisher's Exact Test, *p* = 0.012). Mild *Demodex* density was observed in 72% of acne patients, whereas severe *Demodex* density was present in 54.5% of rosacea patients.

**FIGURE 1:**
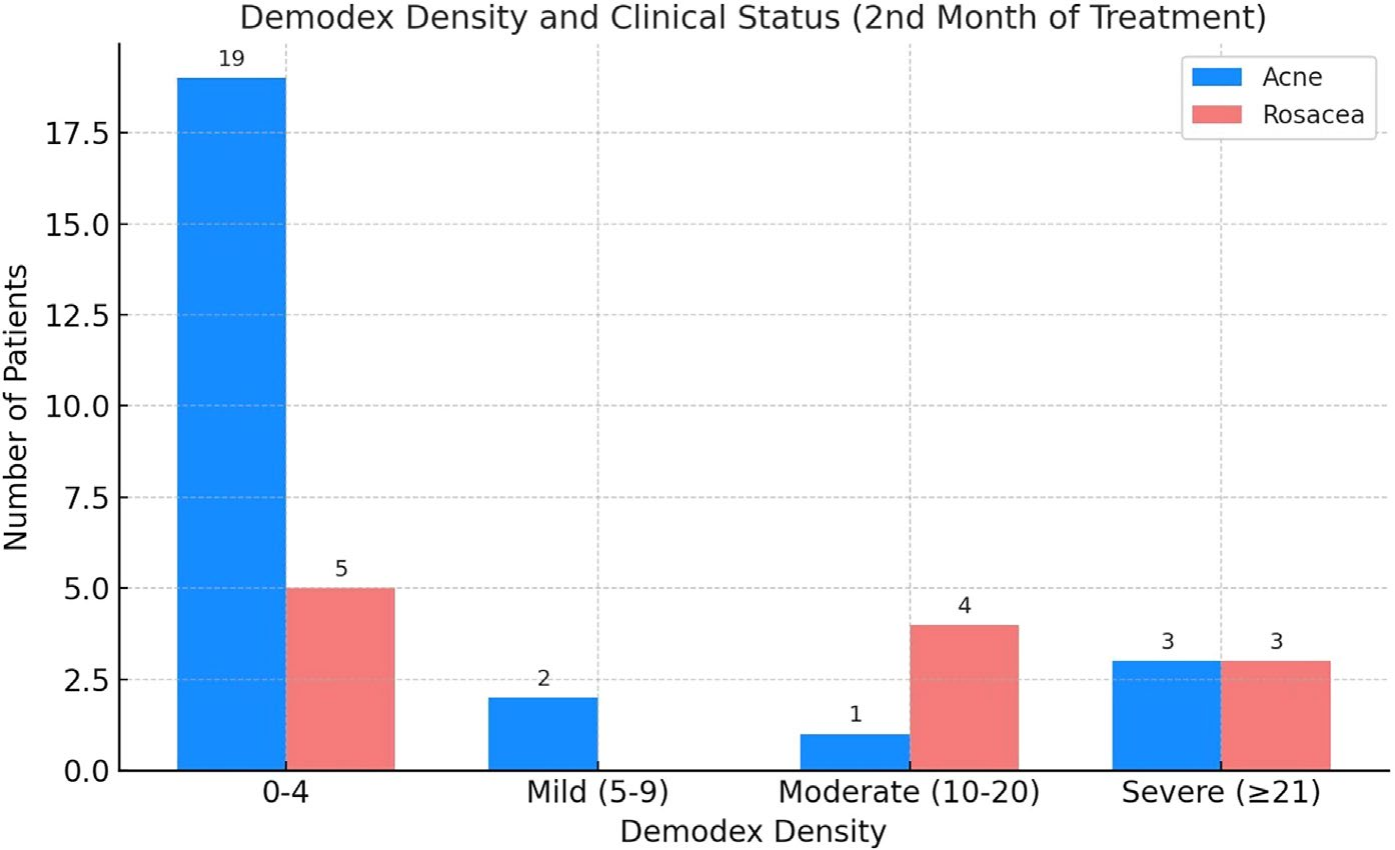
*Demodex* density in the treatment of second month.

By the second month of the treatment period, *Demodex* density had decreased to minimal levels in 76% of acne patients. However, moderate or severe *Demodex* density persisted in 38.4% of rosacea patients (Pearson Chi‐Square test, *p* = 0.022; Fisher's Exact Test, *p* = 0.014) (Figure 1).

**FIGURE 2:**
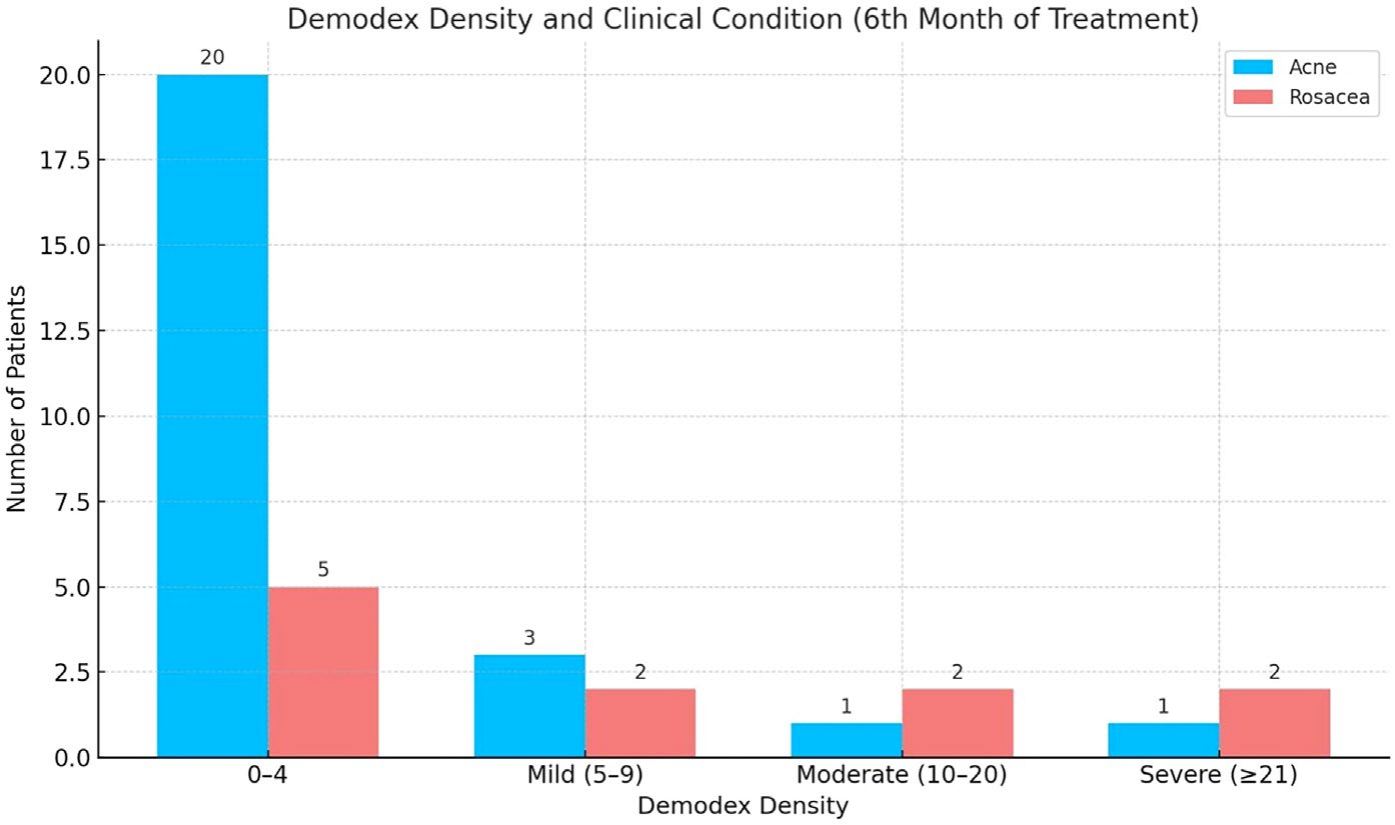
*Demodex* density in the treatment of sixth month.

At the 6‐month treatment mark, a significant reduction in *Demodex* density was observed in acne patients, while moderate and severe density remained in rosacea patients. Throughout the treatment period, acne patients showed a more favorable response, whereas post‐treatment *Demodex* density tended to remain elevated in rosacea patients. However, statistical analyses (Pearson Chi‐Square test, *p* = 0.157; Fisher's exact test, *p* = 0.109) revealed no significant difference in post‐treatment *Demodex* density between acne and rosacea patients (Figure 2).

### 3.4 Change in *Demodex* Density

**FIGURE 3:**
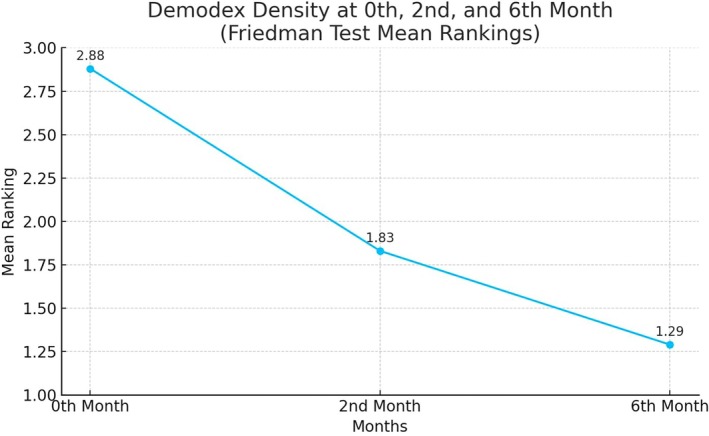
*Demodex* density in the treatment of 0th, second and sixth months (Friedman test mean rankings).

The Friedman test results revealed a significant difference in *Demodex* density between months 0, 2, and 6 (Chi‐Square = 50.864, *p* < 0.001). Analysis of the mean ranks indicated a significant decrease in *Demodex* density over the course of the treatment. Specifically, 25% of patients presented with severe *Demodex* density at baseline (month 0), a proportion that decreased to 8.3% by the sixth month (Friedman test, *p* < 0.05) (Figure 3).

**FIGURE 4:**
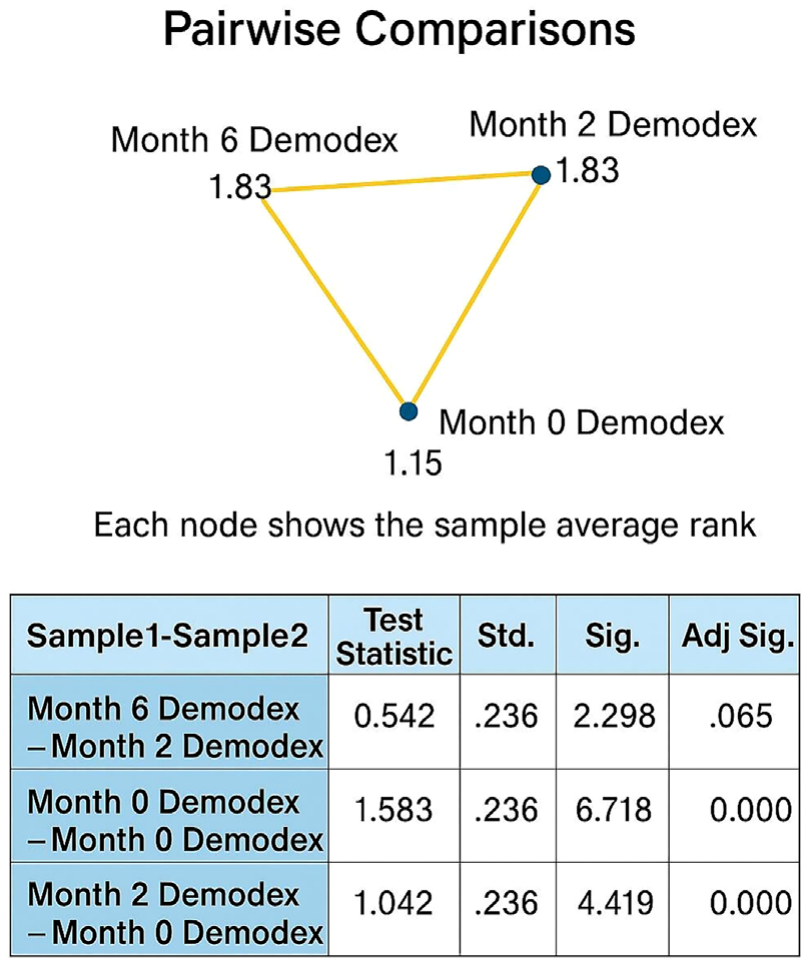
Pairwise comparisons on *Demodex* density in both groups (acne and rosacea patients).

Pairwise comparisons conducted after the Friedman test demonstrated that the treatment had a significant impact on *Demodex* density. Statistically significant differences were observed between the pre‐treatment period (month 0) and both months 2 and 6 (*p* < 0.001). However, no significant difference was found between months 2 and 6 following Bonferroni correction (*p* > 0.05) (Figure 4).

### 3.5 Changes in *Demodex* Density in All Patients (Acne Vulgaris and Rosacea)

The results of the Friedman test indicated a statistically significant difference in *Demodex* density between months 0, 2, and 6 in both acne and rosacea patients. Specifically, in the group of acne patients (*N* = 25), a significant difference was observed (Chi‐Square = 36.622, *p* < 0.001). Similarly, a significant difference was also noted in rosacea patients (*N* = 11) (Chi‐Square = 14.333, *p* = 0.001).

However, despite the significant decrease in *Demodex* density in both groups, the response rate to treatment was higher in acne patients, whereas the reduction in *Demodex* density was relatively lower in rosacea patients.

#### 3.5.1 Acne Patients

**FIGURE 5:**
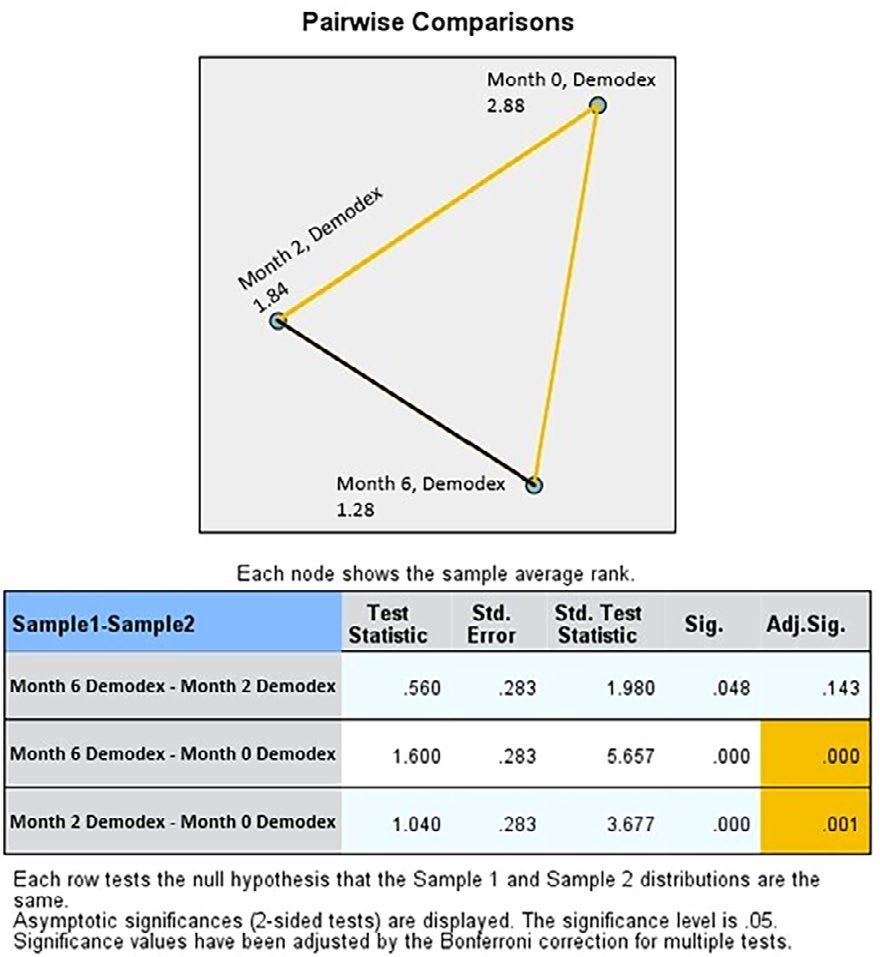
Pairwise comparisons on *Demodex* density in acne patients.

In acne patients, statistically significant differences were found in *Demodex* density between the pre‐treatment measurement (month 0) and months 2 and 6 (*p* < 0.001). However, after applying the Bonferroni correction, no significant difference was observed between months 2 and 6 (*p* > 0.05) (Figure 5).

#### 3.5.2 Rosacea Patients

**FIGURE 6:**
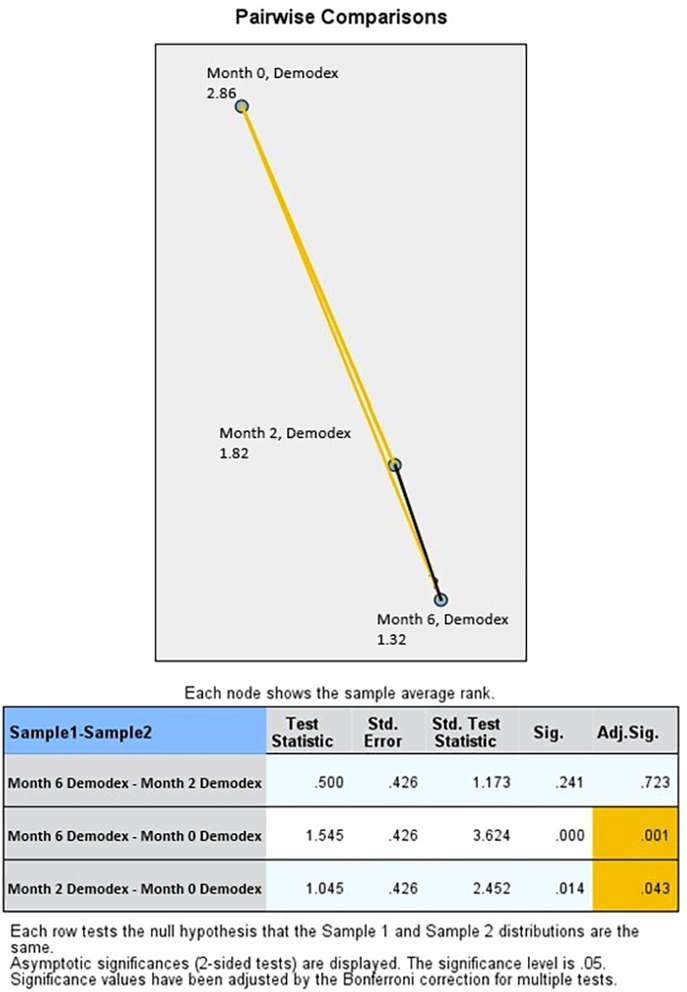
Pairwise comparisons on *Demodex* density in rosacea patients.

Similarly, in rosacea patients, statistically significant differences were observed in *Demodex* density between the pretreatment measurement (month 0) and both months 2 and 6 (*p* < 0.05). However, after Bonferroni correction, the difference between months 2 and 6 was not significant (*p* > 0.05) (Figure 6).

## 4 Discussion

*Demodex* spp. is the most common ectoparasite in humans, and the number of studies on this parasite has been steadily increasing in recent years. While *Demodex* is known to play a significant role in the pathogenesis of rosacea, its involvement in the development of acne vulgaris remains controversial [10, 11]. In a study by Okyay et al. [12], no significant difference was observed in the presence of *Demodex* between acne vulgaris patients and the control group. In contrast, Aktaş Karabay and Aksu Çerman [3] found a statistically significant higher prevalence of *Demodex* positivity in the acne vulgaris group compared to the control group. Furthermore, a meta‐analysis suggested that *Demodex* infestation contributes to the pathogenesis of acne vulgaris [13]. Akçınar et al. [8] indicated that treatment targeting *Demodex* infestation may be beneficial in postadolescent acne patients who are resistant to conventional therapies. The present study is the first to evaluate the impact of isotretinoin treatment on *Demodex* density in patients.

In our study, *Demodex* density was classified using the SYDB method into three categories: mild (5–9 mites/cm^2^), moderate (10–20 mites/cm^2^), and severe (≥ 21 mites/cm^2^). According to this classification, 58.3% of the patients were in the mild group, 16.7% in the moderate group, and 25% in the severe group. A study conducted by Akçınar et al. [8] employed the SYDB method, categorizing *Demodex* density into five groups: 0–5/cm^2^, 5–10/cm^2^, 10–15/cm^2^, 15–20/cm^2^, and > 20/cm^2^. Given that demodicosis is defined as a *Demodex* density exceeding 5/cm^2^ and considering the limited sample size in our study, we found it appropriate to classify the cases into three groups. Furthermore, a study by Zhao et al. [9] utilized the cellophane tape method, classifying *Demodex* density as mild (1–10 mites/5 cm^2^), moderate (11–30 mites/5 cm^2^), and severe (≥ 31 mites/5 cm^2^). Although the cellophane tape method has been used in previous studies, the SYDB method is more frequently preferred today due to its ability to provide fast and reliable results.

According to the results of the Mann–Whitney *U* test in our study, *Demodex* density in rosacea patients was significantly higher than in acne patients during the pre‐treatment period (*p* < 0.01). This finding aligns with the results of Aktaş Karabay and Aksu Çerman's [3] study, where *Demodex* densities were also found to be higher in rosacea patients compared to those with acne. Furthermore, our findings are consistent with the meta‐analysis conducted by Zhao et al. [13], reinforcing the association between elevated *Demodex* levels and rosacea. These results suggest that *Demodex* density could be an important factor in the pathophysiology of rosacea.

*Demodex* spp. thrive in pilosebaceous units, utilizing sebum and cellular proteins as their primary food sources [14, 15]. Literature suggests that cholesterol esters present in sebum create an environment conducive to *Demodex* proliferation [16]. In a study by Sarangua et al. [17], patients with higher *Demodex* folliculorum density exhibited significantly higher sebum levels compared to the control group. Additionally, they observed a correlation between increased sebum secretion, particularly in the forehead region, and the severity of acne [17].

Pierard‐Franchimont et al. demonstrated that sebum levels in patients with mild acne were similar to those in the control group. However, a significant difference in sebum levels was observed in patients with moderate or severe acne compared to the control group [18]. In another study by Clanner‐Engelshofen et al., the effects of drugs influencing sebum production on *Demodex* parasites were examined in an ex vivo setting. The study used retinol and isotretinoin as sebum‐reducing agents, while testosterone and trenbolone were employed as sebum‐stimulating drugs. The results revealed that isotretinoin reduced *Demodex* parasites by 63.4%, proving to be more effective than the other drugs tested [19].

The study by Anon Paichitrojjana (2022) explored the use of a combination of isotretinoin and permethrin in the treatment of ivermectin‐resistant demodicosis. After 2 months of treatment, the patient's initial *Demodex* count of 68/cm^2^ was reduced to 5/cm^2^, demonstrating a significant therapeutic effect [20]. In the present study, the impact of isotretinoin on *Demodex* density was analyzed in detail, revealing a significant decrease in *Demodex* density between the second and sixth months of treatment (*p* < 0.001). The limited sample size may have impacted the statistical power, particularly concerning the nonsignificant findings at the 6‐month mark. Prior to treatment, 58.3% of patients exhibited mild, 16.7% moderate, and 25% severe *Demodex* infestation. However, by the sixth month of treatment, approximately 80% of patients showed a reduction to a *Demodex* density of 0–5/cm^2^. This finding highlights the effectiveness of isotretinoin in reducing *Demodex* density. Furthermore, when analyzing the acne and rosacea clinical groups separately, a significant decrease in *Demodex* density was observed in both groups throughout the treatment period (*p* < 0.001). These results further support the conclusion that isotretinoin is an effective treatment for reducing *Demodex* density, particularly in patients with acne and rosacea.

The results of this study support existing literature indicating that isotretinoin treatment significantly reduces sebum production, thereby decreasing *Demodex* density. In the two‐way comparison conducted with the entire patient population, both the acne and rosacea groups were analyzed separately, and it was determined that the differences between pre‐treatment and the second and sixth months were statistically significant (*p* < 0.001). However, no significant difference was observed between the second and sixth months of treatment (*p* > 0.05). These findings suggest that the sebum‐suppressing effect of isotretinoin is evident within the first 2 months of treatment, leading to a significant decrease in *Demodex* density. It is hypothesized that although sebum production continued to decrease throughout the treatment period, there was no further significant reduction in *Demodex* density between the second and sixth months. A study by Uslu et al. [21] reported that sebum levels in rosacea patients significantly decreased starting from the first month of isotretinoin treatment, with a plateau effect observed throughout the remainder of the treatment. Similarly, Gencebay et al. [22] found a 36% reduction in sebum levels in 36 patients with acne vulgaris compared to baseline. In line with these studies, research by Çölgeçen et al. [23] demonstrated a 74.8% decrease in sebum production by the third month of isotretinoin treatment. The lack of statistically significant differences between groups at the 6‐month follow‐up may be attributed to several factors, including the initially lower *Demodex* density observed in rosacea patients, inherent differences in the pathophysiology of acne and rosacea, and the possibility of varying responses to isotretinoin treatment between these conditions. Our study supports these findings, confirming that isotretinoin treatment reduces sebum levels, which consequently leads to a reduction in *Demodex* density.

## 5 Conclusion

This study makes a significant contribution to the literature as the first to evaluate the effect of isotretinoin treatment on *Demodex* density in patients with acne vulgaris and rosacea. Our findings demonstrate that isotretinoin treatment leads to a significant reduction in *Demodex* counts, which correlates with a decrease in sebum secretion. This result offers valuable insights into the potential mechanism by which isotretinoin may exert its therapeutic effects in *Demodex*‐related dermatological conditions.

## 6 Study Limitations

This study is limited by its relatively small sample size and the absence of a long‐term follow‐up period, which may affect the generalizability and robustness of the findings. Future studies with larger multicenter sample groups and extended follow‐up periods are needed to provide stronger evidence for the effects of isotretinoin on *Demodex* density. Additionally, the inclusion of a control group, allowing for comparison of *Demodex* densities between treated and untreated patients, would strengthen the validity of the results. Clinical symptoms and quality of life assessments could not be consistently evaluated across all participants. Furthermore, future research could explore other mechanisms through which isotretinoin may influence *Demodex* density, beyond its effects on sebum suppression.

## Author Contributions

A.M.J.C., E.T.A., A.Y. performed the research. A.M.J.C., E.T.A., A.Y. designed the research study. C.T.A. and S.D.P. contributed essential reagents or tools. A.M.J.C., E.T.A., A.Y. analyzed the data. A.M.J.C., E.T.A., A.Y. wrote the paper.

## Ethics Statement

Ethical approval for the thesis was obtained from the Mugla Sıtkı Kocman University Ethics Committee, with decision number 123, dated December 21, 2023.

## Conflicts of Interest

The authors declare no conflicts of interest.

## Data Availability

The data that support the findings of this study are available from the corresponding author upon reasonable request.
